# Subcortical brain mapping strategies for intraoperative identification of the optic radiation during asleep and awake surgery: a scoping review

**DOI:** 10.1016/j.bas.2026.106175

**Published:** 2026-07-14

**Authors:** Marco Obersnel, Pablo Alvarez-Abut, Sivani Sivanrupan, Andreas Raabe, Philippe Schucht, Kathleen Seidel

**Affiliations:** Department of Neurosurgery, Inselspital, Bern University Hospital, University of Bern, Rosenbühlgasse 25, Bern, 3010, Switzerland

**Keywords:** Optic radiation, Visual evoked potentials, Intraoperative neurophysiological monitoring, Brain mapping, Subcortico-cortical evoked potentials, Neuro-oncology

## Abstract

**Introduction:**

Postoperative visual deficits after glioma surgery may significantly reduce quality of life. Multiple techniques for intraoperative mapping of the optic radiation (OR) have been described, but no consensus exists regarding optimal strategies, particularly for novel neurophysiological recording techniques.

**Research question:**

We conducted a scoping review to systematically organize the existing paradigms for intraoperative OR mapping, distinguishing awake task-based approaches from evoked potential recording techniques, and to outline future directions for standardization.

**Material and methods:**

This scoping review was conducted following the PRISMA-ScR guidelines. Studies describing intraoperative mapping of the OR in any surgical setting were included. Stimulation and recording parameters, as well as intraoperative task paradigms, were extracted and synthesized.

**Results:**

Nineteen studies involving 212 patients were included: four reporting neurophysiological recording techniques, 14 describing awake mapping approaches, and one combining both. Three main technique categories were identified: subcortico-cortical evoked potentials (SCEP), subcortical recorded visual evoked potentials (sVEP), and awake task-based mapping.

**Discussion and conclusion:**

A modified picture-naming task during awake mapping, presenting two figures diagonally on a screen, has proven useful in estimating the OR boundary, allowing maximal safe resection while limiting postoperative deficits to an acceptable quadrantanopia in most low-grade glioma surgeries. Alternative awake paradigms show promise but require further validation. Intraoperative neurophysiological recording techniques are technically feasible, and their role could be relevant in the future, particularly for patients unsuitable for awake surgery. However, methodological standardization and clinical validation are needed.

## Highlights

• Intraoperative mapping of optic radiation lacks standardized approaches.
• Optic radiation mapping has been described in both awake and asleep surgery.
• Awake mapping may allow maximal safe resection with acceptable visual field outcomes.
• Novel evoked potential recording techniques show potential for asleep mapping.

## 1 Introduction

Vision is considered one of the most highly valued senses in humans (Enoch et al., 2019), and even partial impairment can lead to a significant reduction in the quality of life. As for the motor and language systems, it is important to prevent or minimize injuries to the visual pathways during neurooncological surgery. Visual deficits such as quadrantanopia or hemianopia may compromise many professional activities, daily life function and, equally importantly, the ability to drive.

Intraoperative monitoring of the optic radiation (OR) through flash visual evoked potentials (VEP) has long been described (Sokol, 1976; Soumpasis et al., 2023). Although it is considered less reliable than motor evoked potentials monitoring, it is widely used in clinical practice. However, while monitoring provides functional surveillance and carries important predictive value for postoperative outcomes, intraoperative mapping is the tool that may guide surgical decision-making by identifying eloquent tissue and defining functional boundaries in real time.

Mapping for the integrity of the motor pathways can be reliably performed under general anesthesia using direct cortical or subcortical stimulation with electromyographic (EMG) recording (Asimakidou et al., 2021; Gerritsen et al., 2026; Seidel et al., 2025). In contrast, mapping for language function traditionally relies on awake mapping, with a low-frequency bipolar stimulation (Penfield paradigm) during language task execution (Sanai et al., 2008; Duffau et al., 2002, 2008; Szelényi et al., 2010). More recently, cortico-cortical evoked potentials (CCEPs) and subcortico-cortical evoked potentials (SCEPs) have emerged as promising adjuncts. Although they are not yet fully clinically validated, they may enable monitoring and mapping of associative pathways in patients who are not candidates for awake surgery (Matsumoto et al., 2004; Seidel et al., 2024; Wasserman et al., 2021; Yamao et al., 2021; Rossel et al., 2023).

Similarly, mapping for the OR has been described both in awake and asleep settings (Santos et al., 2022). However, no clear consensus has emerged regarding the optimal stimulation strategies and methods for assessing functional responses, whether evoked potential or task-based evaluation (Gerritsen et al., 2026). In recent years, novel recording techniques and stimulation strategies have been proposed, yielding heterogeneous and not always comparable results.

The aim of this review is therefore to (1) systematically organize and critically investigate the existing paradigms for intraoperative mapping of the OR, distinguishing between awake task-based approaches and evoked potential recording techniques, and to (2) outline future directions for methodological standardization and clinical integration.

## 2 Material and methods

### 2.1 Study design

This scoping review was conducted in accordance with the Preferred Reporting Items for Systematic Reviews and Meta-Analyses extension for Scoping Reviews (PRISMA-ScR) guidelines (Tricco et al., 2018).

### 2.2 Search strategy

A comprehensive literature search was performed in the electronic databases PubMed (MEDLINE), Scopus, Embase, CINAHL, and the Cochrane Library. Databases were searched from inception to February 2026. The search strategy combined vocabulary related to the OR and visual pathway, including “optic radiation”, “optic tract”, and “visual pathway,” with terms associated with intraoperative mapping, including “intraoperative”, “mapping”, “recording”, “subcortical stimulation”, “subcortico-cortical evoked potentials”, and “axono-cortical evoked potentials”. Boolean operators and tumor-related terms, including “tumor” and “glioma”, were used to refine the search. The full electronic search strategy is provided in the Supplementary Material. The reference lists of retrieved articles and the sets of similar articles suggested by the database were screened in order to identify additional relevant citations.

### 2.3 Eligibility criteria

Studies were considered eligible if they described intraoperative mapping of the OR and provided a methodological description of the mapping technique, including stimulation parameters and/or task paradigms. Studies reporting VEPs were included only when recordings were obtained from subcortical electrodes placed adjacent to the optic radiation. Studies conducted in animal models, purely anatomical or radiological investigations without intraoperative mapping, review articles, and studies reporting monitoring alone without active mapping were excluded. Accordingly, studies limited to VEP recordings from scalp corkscrews or subdural cortical strip electrodes were excluded, as the present review focused on active intraoperative mapping of the optic radiation rather than monitoring of the visual pathway. No restrictions were applied regarding language or year of publication.

### 2.4 Study selection

All retrieved records were exported to reference management software, and duplicate entries were removed. Titles and abstracts were independently screened by two reviewers. Full-text articles were subsequently assessed for eligibility by the same reviewers. Disagreements were resolved through discussion with a third reviewer.

### 2.5 Data charting process

Data were charted using a predefined Microsoft Excel (Microsoft 365; Microsoft Corporation, Redmond, WA, USA) spreadsheet developed specifically for this review.

The following variables were extracted: author and year of publication, study design, number of patients, number of positive mapping cases, underlying pathology (tumor, vascular, epilepsy), type of mapping, stimulation parameters and intensity, recording modality or task paradigm, reported neurophysiological or clinical responses, distance from the OR when available, and additional methodological notes.

### 2.6 Synthesis of results

The extracted data were organized into two principal methodological categories: awake task-based mapping approaches and evoked potential recording techniques. Findings were structured and summarized in tabular form to facilitate comparison across studies and to identify areas of methodological similarities and divergence.

## 3 Results

### 3.1 Study selection and general characteristics

The database search yielded 1095 records, including 373 from PubMed, 306 from Scopus, 185 from Embase, 221 from CINAHL, and 10 from the Cochrane Library. After removal of 652 duplicates, 443 records were screened. Following title and abstract screening, 23 reports were sought for retrieval; two could not be retrieved because only abstracts were available, and 21 were assessed for eligibility. Seventeen studies met the inclusion criteria.

**Fig. 1:**
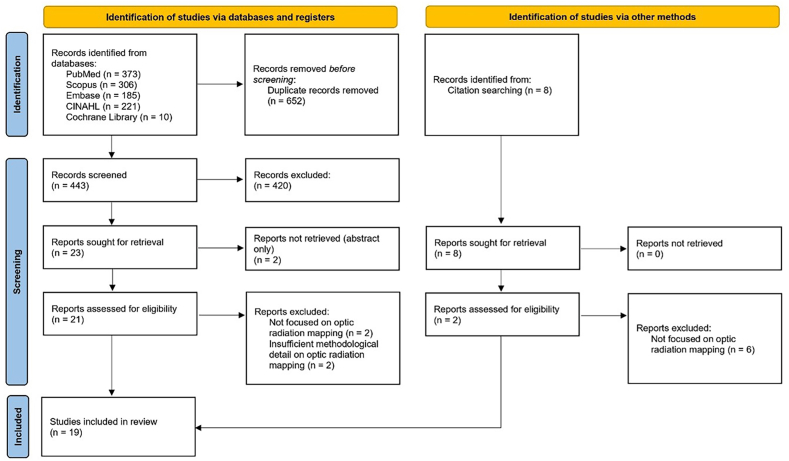
Flow diagram for study selection.

In addition, eight records were identified through citation searching. After full-text assessment, six were excluded, resulting in two additional studies included in the final analysis. Overall, 19 studies were included: four reporting evoked potential recording techniques, 14 describing awake mapping approaches, and one combining both methodologies. All included studies were published in peer-reviewed journals. The study selection process is illustrated in Fig. 1.

### 3.2 Summarized analysis

Overall, the included studies involved 212 patients, of whom 183 underwent awake mapping and 40 underwent subcortical mapping with evoked potential recording techniques; 11 patients underwent both.

The underlying pathology was heterogeneous, predominantly comprising gliomas, with a smaller number of metastatic (n = 3), vascular (n = 2), and epilepsy (n = 4) cases. Evoked potential recording techniques were mainly applied in patients with high-grade gliomas (n = 28), whereas only four patients had low-grade gliomas. Awake mapping was more frequently performed in patients with low-grade gliomas than in patients with high-grade gliomas (n = 110 vs n = 72).

Formal comparative analyses and pooled outcome estimates were not performed because of substantial heterogeneity in study design, underlying pathology, and outcome reporting, as well as the limited number of patients within each subgroup. Nevertheless, a descriptive classification was undertaken. Based on the site and modality of stimulation, recording location, and type of functional response assessed, three recurring methodological strategies were identified: (1) subcortico-cortical evoked potentials (SCEPs), involving direct subcortical stimulation of the optic radiation with recording from the calcarine cortex; (2) subcortical recorded visual evoked potentials (sVEPs), involving flash stimulation with recording from the optic radiation; and (3) awake task-based mapping, involving direct subcortical stimulation during visual task performance or assessment of stimulation-induced visual phenomena.

SCEP recording was reported in four studies involving 26 patients, sVEP recording in two studies involving 15 patients, and awake task-based mapping in 15 studies involving 183 patients; one patient underwent both SCEP and sVEP recording. Positive responses were reported in 145 awake-mapping cases, 20 SCEP cases, and 12 sVEP cases.

**Fig. 2:**
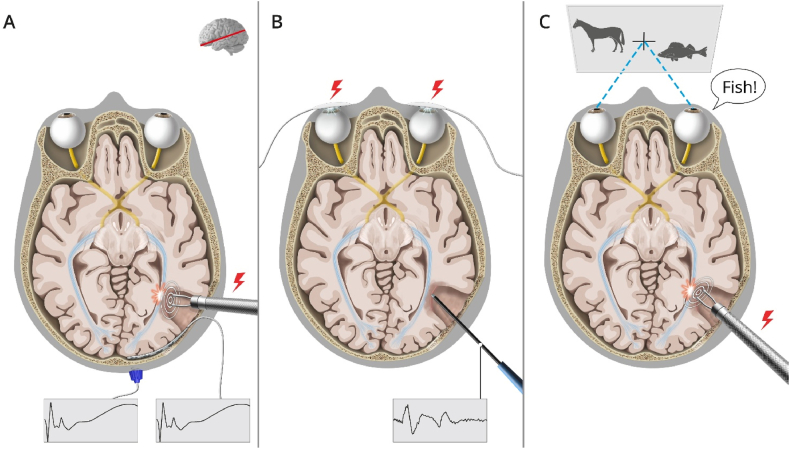
The three main strategies for optic radiation (OR) mapping are represented. A diagonal cut corresponding to the orientation of the OR has been chosen to enhance the relationship between the OR and the lateral ventricles. (**A**) **Subcortico-cortical evoked potential (SCEP).** Subcortical stimulation is performed with a bipolar probe in the resection cavity of a parieto-occipital tumor. Recordings of the SCEPs are taken from the occipital cortex using either a subdural strip electrode or a transcranial corkscrew electrode. (**B**) **Subcortical recorded visual evoked potential (sVEP).** Flash light stimulation is provided with light-emitting diodes (LED) goggles as in conventional visual evoked potentials (VEPs). Recordings of the sVEPs are taken from the floor of the surgical cavity using a monopolar probe, with a reference at FPz (not visible in the image). (**C**) **Awake mapping.** The modified picture-naming task is represented. Stimulation is performed in the floor of the surgical cavity while the patient is asked to name the provided pictures, maintaining gaze fixed on the central cross. Stimulation of the OR prevents the patient from seeing the contralateral quadrant, so the patient names only the ipsilateral picture, indicating a selective visual field disturbance rather than a language deficit. Note: The illustrated recording traces in panels A and B were inspired by Zauli et al., 2024, and Shahar et al., 2017, respectively. Due to the heterogeneity of the reported results, universal waveform patterns cannot yet be defined.

The three strategies are described in Sections 3.3, 3.4 and illustrated in Fig. 2.

### 3.3 Evoked potential recording techniques: SCEP and sVEP

Table 1 summarizes the studies describing evoked potential recordings. Two principal techniques were identified. The first involves the recording of SCEPs from the occipital cortex using either a subdural strip electrode (Zauli et al., 2024; Rajashekar et al., 2022) or transcranial corkscrew electrodes (Boëx et al., 2021; Baş et al., 2023) with subcortical stimulation of the white matter in proximity to the OR. Subcortical stimulation was generally performed with a bipolar probe, biphasic pulse, with frequency 1-4 Hz and variable stimulation intensity from 5 to 20 mA.

**Table 1:** Overview of included studies describing evoked potential recording techniques.

Study characteristics					Stimulation protocol				Outcome		Notes
Authors	Type	N patients	N positive responses	Pathology	Type of mapping	Stimulation parameters	Intensity	Recording	Curve characteristics	Distance from OR (DTI) responses	
Shahar et al., 2017	Retrospective case series.	13	10	HGG (10), LGG (2), metastasis (1).	Subcortical recording (awake surgery).	Binocular flash stimulation with LED embedded in goggles (pulse width 15 μs, frequency 1.2 Hz).	-	Bayonet-style monopolar probe near the optic radiation. Bandwidth 1–300 Hz. Reference in FPz.	Similar to cortical VEP (N75 and P100 peaks). No phosphenes reported.	0–9.3 mm (tumor-to-optic radiation distance).	
Zauli et al., 2024	Retrospective case series.	2	2	Drug-resistant focal epilepsy (2).	Subcortical recording (asleep surgery).	LED goggles delivering simultaneous binocular red flashes, frequency 1 Hz.	3 cd s/m^2^	Multicontact SEEG intracerebral electrode near/in the optic radiation (with neuronavigation guidance): diameter: 0.8 mm; contact length: 2 mm, inter-contact distance: 1.5 mm.	Latency 50.2–57.7 ms, amplitude 3 μV. Initial small positive deflection followed by short burst of sinusoidal waves with a frequency of 100-150 Hz.	1.5–3.5 mm (distance between the recording contact and the intra-parenchymal contact closest to the bottom of the cavity).	
			1	Drug-resistant focal epilepsy (1).	SCEP.	Multicontact SEEG intracerebral electrode in the optic radiation (with neuronavigation guidance): diameter: 0.8 mm; contact length: 2 mm, inter-contact distance: 1.5 mm.	-	4- or 6-contact strip electrode on the calcarine cortex (position finalized when VEP had highest amplitude and definition).	Latency 5.6 ms.	-	The sum of subcortical recording (50.2 ms) and SCEP (5.6 ms) latencies approximates the flash VEP latency recorded from the calcarine cortex (63.4 ms).
Boëx et al., 2021	Prospective case series.	9	5	HGG (5), cavernoma (1), AVM (1), HPC sclerosis (1).	SCEP.	Bipolar probe, biphasic, 0.2 ms per phase, frequency 1.3 Hz. 30 stimulations on average.	5–8 mA	Bilateral central, parietal, parieto-occipital, and occipital subcutaneous corkscrew electrodes. Bandpass filtering: 5–4000 Hz.	Latency P1 2.4–5.4 ms, P2 15–21.6 ms (only in 3 patients with a distance <2.1 mm).	<4.5 mm.	
Rajashekar et al., 2022	Prospective case series.	15	13	HGG (13), metastasis (1), LGG (1).	SCEP.	Ball tip bipolar fork probe, 8 mm tip spacing, frequency 1–4 Hz.	Mean 12 ± 2.3 mA (max 20 mA)	Strip perpendicular to calcarine fissure.	P2 and N3 peaks were measured at baseline and during debulking. Results not reported.	3.56 ± 5.22 mm.	Study not focused on SCEP.
Baş et al., 2023	Case report.	1	1	LGG (1).	SCEP.	Bipolar probe, biphasic, 0.2 ms per phase, frequency 1.3 Hz.	3.5 mA	Bilateral occipital and parietal subcutaneous corkscrew electrodes	Latency P1 5–10 ms, P2 40 ms.	3 mm.	

Abbreviations: cd, candela; DTI, diffusion tensor imaging; HGG, high-grade glioma; LED, light-emitting diode; LGG, low-grade glioma; OR, optic radiation; SCEP, subcortico-cortical evoked potentials; SEEG, stereo electroencephalography; sVEP, subcortical recorded visual evoked potentials; VEP, visual evoked potentials.

The second approach consisted of recording sVEP through a stereo-electroencephalographic (SEEG) electrode or a monopolar probe placed near the OR, using flash light stimulation as in conventional VEPs (pulse width 15 μs, frequency 1-1.2 Hz), in awake (Shahar et al., 2017) or asleep patients (Zauli et al., 2024).

Both techniques were applied in limited cohorts: SCEP recording was performed in 26 patients and sVEP recording in 15 patients, with one patient undergoing both.

### 3.4 Awake mapping techniques

Table 2 summarizes the awake mapping techniques. Following the initial report by Duffau et al. (2004), in which visual disturbances were observed during subcortical stimulation performed in conjunction with a standard picture-naming task, several structured task paradigms were subsequently developed.

**Table 2:** Overview of included studies describing awake mapping techniques.

Study characteristics					Stimulation protocol				Outcome		Notes
Authors	Type	N patients	N positive responses	Pathology	Type of mapping	Stimulation parameters	Intensity	Task	Reported symptoms	Distance from OR (DTI) responses	
Duffau et al., 2004	Case report.	1	1	LGG (1).	Awake mapping.	Bipolar probe (5 mm tip spacing), biphasic, frequency 60 Hz, single pulse, pulse width 1 ms. Stimulations repeated 6 times without informing the patient, during picture naming.	5 mA	-	Shadow in left superior visual field and picture distortion.	-	First report of awake mapping of the OR.
Nguyen et al., 2011	Case report.	1	1	HGG (1).	Awake mapping.	Bipolar stimulation.	5 mA	White screen with central fixation; red targets (test) vs green (control). Patient reported fading of red stimuli. Additional kinetic laser testing and spot checks across vertical meridian.	Bright flashing lights in the right visual field.	-	
Šteňo et al., 2012	Case report.	1	1	LGG (1).	Awake mapping.	Bipolar stimulation.	4 mA	Dog drawing with central fixation cross. The operator used a laser pointer to indicate different parts of the image and asked for verbal confirmation of the localization.	Bright phosphenes that did not allow visualization of parts of the dog's body.	-	
Gras-Combe et al., 2012	Prospective case series.	14	14	LGG (12), HGG (2).	Awake mapping.	Bipolar probe (5 mm tip spacing), biphasic, frequency 60 Hz, single pulse, pulse width 1 ms, stimulation duration 4 s.	2–4 mA	Modified picture-naming task with 2 pictures placed diagonally on the screen.	Blurred vision, phosphenes, shadows.	-	This task became the most widely used paradigm for awake OR mapping.
Fernández Coello et al., 2013	Case report.	1	1	LGG (1).	Awake mapping.	Low frequency stimulation (4 errors for 4 trials).	2 mA	Modified picture-naming task with 2 pictures placed diagonally on the screen.	Transient visual hemiagnosia during stimulation, resolving after cessation.	-	
Chan-Seng et al., 2014	Retrospective case series.	8	5	LGG (8).	Awake mapping.	Bipolar probe (5 mm tip spacing), biphasic, frequency 60 Hz, single pulse, pulse width 1 ms, stimulation duration 4 s.	1.5–3 mA	Modified picture-naming task with 2 pictures placed diagonally on the screen.	Blurred vision, phosphenes or visual illusions.	-	
Šteňo et al., 2014	Case report.	1	1	LGG (1).	Awake mapping.	Bipolar stimulation.	4 mA	Dog drawing with central fixation cross. The operator used a laser pointer to indicate different parts of the image and asked for verbal confirmation of the localization. With eyes covered, patient was instructed to report any phosphenes.	Quadrantic phosphenes, more evident with eyes covered.	-	
Sarubbo et al., 2014	Case series.	3	3	LGG (2), HGG (1).	Awake mapping.	Bipolar stimulator (0.7 mm tip spacing).	Stimulation threshold was determined by eliciting complete speech arrest at vPMC	Modified picture-naming task with 2 pictures placed diagonally on the screen.	Flashes.	-	
Mazerand et al., 2017	Case report.	1	1	HGG (1).	Awake mapping.	Subcortical stimulation.	6 mA	Modified Esterman test using FEX-VRH (virtual reality headset): during stimulation, an orthoptist delivers visual light stimuli via the headset and records the patient's responses.	No visual symptoms during stimulation.	-	
Shahar et al., 2017	Retrospective case series.	14	5	HGG (11), LGG (2), metastasis (1).	Awake mapping.	Bayonet-style monopolar probe in the OR. 2 s trains at 50 Hz (500 μs pulse width) with 5 s inter-train interval. Return subdermal needle in the subcutaneous tissue far from the occipital lobe.	3–11.5 mA	-	Phosphenes in the contralateral visual field.	2–7 mm (tumor-to-OR distance).	Positive correlation between subcortical stimulation threshold and tumor-to-OR distance; no response when the tumor was within or ≤1 mm from the OR.
Rolland et al., 2018	Prospective case series.	14	6	LGG (12), HGG (2).	Awake mapping.	Bipolar probe (5 mm tip spacing), biphasic, frequency 60 Hz, single pulse, pulse width 1 ms, stimulation duration 4 s.	1.5–3 mA	Modified picture-naming task with 2 pictures placed diagonally on the screen.	Blurred vision, phosphenes, shadows.	-	
Conti Nibali et al., 2020	Retrospective double-arm.	54	54	HGG (24), LGG (30).	Awake mapping.	Low frequency stimulation.	Lowest current intensity producing interferences when stimulating the vPMC	Intraoperative Visual Field Task (iVT): random presentation of three different numbers, one at a time, in the extreme left or right hemifield while the patient fixates centrally and is asked to name the number.	Detection error (inability to perceive the number); discrimination error (hesitation or incorrect number identification).	-	OR stimulation predominantly induced detection errors, whereas ILF stimulation induced discrimination errors.
		38	38	HGG (20), LGG (18).	Awake mapping.	Low frequency stimulation.	Lowest current intensity producing interferences when stimulating the vPMC	Modified picture-naming task with 2 pictures placed diagonally on the screen.	Naming errors.	-	
Berro et al., 2021	Retrospective case series.	17 (18 surgeries)	13	LGG (17).	Awake mapping.	Bipolar probe (5 mm tip spacing), biphasic, frequency 60 Hz, single pulse, pulse width 1 ms, stimulation duration 4 s.	1.5–3.25 mA	Modified picture-naming task with 2 pictures placed diagonally on the screen.	Quadrantanopia, phosphenes, blurred vision, hemianopia.	-	
Sangrador-Deitos et al., 2022	Case report.	1	1	LGG (1).	Awake mapping.	Bipolar probe (5 mm tip spacing), biphasic, pulse frequency 60 Hz, single pulse, pulse width 1 ms, stimulation duration 4 s.	1–5 mA	Modified picture-naming task with 2 pictures placed diagonally on the screen.	Flashes.	-	
Ichinose et al., 2022	Retrospective case series.	14	14	HGG (10), LGG (4).	Awake mapping.	Bipolar probe, biphasic, frequency 60 Hz, pulse width 0.2 ms.	2-6 mA	Modified picture-naming task with 2 pictures placed diagonally on the screen.	Phosphenes (85%), blurred vision (12%), or hallucinations (3%).	0–9 mm.	

Abbreviations: DTI, diffusion tensor imaging; HGG, high-grade glioma; ILF, inferior longitudinal fasciculus; LGG, low-grade glioma; OR, optic radiation; vPMC, ventral premotor cortex.

Gras-Combe et al., in 2012 (Gras-Combe et al., 2012), described the “modified picture-naming task”, that became the most commonly used in following papers (n = 110) (Fernández Coello et al., 2013; Chan-Seng et al., 2014; Sarubbo et al., 2014; Rolland et al., 2018; Conti Nibali et al., 2020; Berro et al., 2021; Sangrador-Deitos et al., 2022; Ichinose et al., 2022). Other authors proposed alternative tasks, with targets differentiated by colors and dynamic testing (n = 1) (Nguyen et al., 2011), with a single figure and laser indication (n = 2) (Šteňo et al., 2012; Šteňo et al., 2014), or more high-tech approaches such as an automated appearance of targets in the monitor (n = 54) (Conti Nibali et al., 2020) or even using a virtual reality headset (n = 1) (Mazerand et al., 2017).

In almost all studies, stimulation was performed with a bipolar probe delivering a biphasic current, with frequency 60 Hz, single pulse, pulse width 1 ms and 4 s duration (Penfield stimulation paradigm). Stimulation was generally performed multiple times during the task with real and sham stimulation. Stimulation intensity was variable from 1.5 to 6 mA.

Shahar et al., 2017, who is present in both tables, used both awake mapping while stimulating the OR and recording of sVEP. However, they did not describe a specific task (Shahar et al., 2017). Furthermore, this is the only article in which subcortical stimulation was performed with a monopolar probe.

The modified picture-naming task became the most widely adopted paradigm, being used in 9 of 15 studies. Among these studies, which shared a comparable methodology and included a relatively large number of patients (n = 110), clinical outcomes could be meaningfully analyzed (Table 3). A total of 92 patients were identified, most of whom had no preoperative visual deficits (6/79, 7.5% of patients with available preoperative clinical data). At follow-up (ranging from one to three months across studies), 23% of patients presented no postoperative visual deficits, 68% developed an (expected) quadrantanopia, and 8% developed homonymous hemianopia.

**Table 3:** Overview of clinical outcomes in studies using the modified picture-naming task.

Authors	N positive mapping	Preoperative deficit	Postoperative deficit at follow-up	EOR %	EOR absolute
Gras-Combe et al., 2012	14	0	No deficit (1), quadrantanopia (12), homonymous hemianopia (1).	GTR (3), STR (11).	Complete (3), near-total (6), subtotal (5).
Fernández Coello et al., 2013	1	0	Quadrantanopia (1).	GTR (1).	Complete (1).
Chan-Seng et al., 2014	5	0	Quadrantanopia (5).	GTR (1), STR (4).	Complete (1), near-total (2), subtotal (2).
Sarubbo et al., 2014	3	0	Quadrantanopia (3).	GTR (3).	Complete (3).
Rolland et al., 2018	6	0	No deficit (2), quadrantanopia (4).	NA	NA
Conti Nibali et al., 2020	38	NA	No deficit (10), quadrantanopia (24, of which 12 symptomatic), hemianopia (4).	GTR (29), STR (6), PR (3).	NA
Berro et al., 2021	13	0	No deficit (4), quadrantanopia (9).	GTR (4), STR (13).	Complete (4), near-total (4), subtotal (8), partial (1).
Sangrador-Deitos et al., 2022	1	Quadrantanopia (1).	Quadrantanopia (1).	STR (1).	Subtotal (1).
Ichinose et al., 2022	11	Quadrantanopia (5).	No deficit (4), quadrantanopia (5), homonymous hemianopia (2).	NA	NA

Abbreviations: EOR, extent of resection; GTR, gross total resection; NA, not available; PR, partial resection; STR, subtotal resection.

Studies using tasks other than the modified picture-naming provided less detailed descriptions of stimulation parameters, reporting only the use of bipolar low-frequency stimulation (see Table 2 for a complete overview).

## 4 Discussion

In this review, we systematically examined the available literature for subcortical brain mapping strategies to intraoperatively identify the OR. The following sections discuss these techniques, summarizing the current state of the art and highlighting their potential advantages and current limitations.

### 4.1 Historical and anatomical considerations

The OR was first described in 1857 by Louis Pierre Gratiolet in his seminal work “Anatomie comparée du Système Nerveux Considéré dans ses rapports avec l’Intelligence“ (Comparative anatomy of the nervous system considered in relation to intelligence) in Paris (Peltier et al., 2006). Two years earlier, in 1855, Bartolomeo Panizza had already demonstrated the role of occipital cortex in vision through animal and post-mortem human studies, as reported in his work “Osservazioni sul nervo ottico”(Observations on the optic nerve) (Zago et al., 2000). In 1918 the Irish neurologist Gordon Holmes published a case series with gunshot injuries involving the OR, correlating these lesions with homonymous hemianopia (Holmes, 1918).

Nowadays, the anatomy of the OR is well established, largely owing to white matter dissection studies (Peltier et al., 2006; Rubino et al., 2005; Párraga et al., 2012) and diffusion tensor imaging (DTI) (Powell et al., 2005; Sherbondy et al., 2008).

After leaving the lateral geniculate body, the OR fibers are classically divided into three main bundles. The anterior bundle runs within the roof of the temporal horn of lateral ventricle, then turns backward forming the Meyer's loop and continues along the lateral surface of the temporal horn, below the atrium and the occipital horn, terminating in the lower lip of the calcarine fissure. The central bundle, after crossing the roof of the temporal horn, courses along the lateral wall of the atrium and occipital horn. The upper (or posterior) bundle passes more directly around the atrium and posterior horn, reaching the upper lip of the calcarine fissure (Peltier et al., 2006; Rubino et al., 2005). Together with the tapetum medially and associative fibers laterally, particularly the inferior fronto-occipital fasciculus (IFOF) and the middle longitudinal fasciculus (MLF), the OR constitutes part of the sagittal stratum (Yagmurlu et al., 2015).

DTI studies have largely confirmed cadaveric findings (Peltier et al., 2006; Ebeling and Reulen, 1988). By placing a region of interest (ROI) in the posterolateral thalamus and another in the calcarine cortex, the OR can be reconstructed and integrated into preoperative surgical planning and intraoperative neuronavigation workflows (Sherbondy et al., 2008; Shi et al., 2022; Faust and Vajkoczy, 2016).

However, due to its changes in direction, combined with the presence of crossing and kissing fibers, the reconstruction of the OR remains a significant challenge for deterministic fiber tracking approaches (Martínez-Heras et al., 2015). These methods often struggle to accurately resolve complex fiber configurations, particularly in regions where multiple fibers with different orientations coexist within a single voxel. In contrast, probabilistic fiber tracking techniques, especially those based on spherical deconvolution or Q-ball imaging, have been shown to better capture the underlying fiber architecture. By modeling multiple fiber orientations within each voxel, these approaches provide a more robust and accurate reconstruction of intricate white matter pathways such as the OR (Lim et al., 2015; Lenga et al., 2024). Anyhow the remaining challenge in all fiber tracking techniques is tumor associated edema (Filipiak et al. 2026). Edema, in fact, leads to a marked reduction in fractional anisotropy (FA), causing premature termination of the fibers.

A simplified interactive three-dimensional model showing the relationship between the optic pathways and the ventricular system is provided in the Supplementary Material.

### 4.2 Evoked potential recording techniques

Two approaches were described: the SCEP, consisting of the stimulation of OR and recording from calcarine cortex, and the sVEP, consisting of the flash stimulation of the eye and recording from the OR.

#### 4.2.1 Subcortico-cortical evoked potentials (SCEP)

Four studies described the recording of SCEP during stimulation of the OR: two case reports and two case series. Stimulation parameters were not consistently reported across studies. The most recurrent paradigm consisted in biphasic stimulation using a bipolar probe, with a pulse duration of 0.2 ms per phase and a frequency of 1.3 Hz; stimulation intensity ranged from 3.5 mA to 20 mA through the different studies. Results were too heterogeneous and the sample size too small to establish a correlation between stimulation intensity and the distance between the stimulation site and the OR.

Recordings were obtained using subcutaneous corkscrew electrodes placed over occipital and parietal regions by Boëx et al. and Baş et al. (Boëx et al., 2021; Baş et al., 2023). Across the three available recording figures (two from Boëx et al. and one from Baş et al.), three slightly different corkscrew recording montages were observed (Supplementary material). In contrast, Zauli et al. and Rajashekar et al. used 4- or 6- contact strips positioned directly over the exposed calcarine cortex (Zauli et al., 2024; Rajashekar et al., 2022).

The recorded waveforms were heterogeneous across studies. However, either one or two positive peaks with anyhow very variable latencies were reported. Rajashekar et al. did not report detailed recording data or figures (Rajashekar et al., 2022). Zauli et al. reported a P1 peak with a latency of 5.6 ms in one patient, with apparent reproducibility across two recordings; although not described in the text, a P2 peak with the latency of 8-9 ms can be identified in the figure (Zauli et al., 2024). Boëx et al. described two peaks: P1 (latency 2.4-5.4 ms), recorded in five patients, and an additional P2 (latency 15-21.6 ms), recorded in three patients (Boëx et al., 2021). P2 was observed only when stimulation was performed very close to the OR (<2.1 mm). This association warrants further investigation, as one of the two patients in whom only P1 was recorded had a stimulation site directly on the OR without generating P2, suggesting that proximity alone may not fully explain P2 generation. However, waveform reproducibility was limited, and differentiation of peaks, particularly P2, from background activity and prolonged artifacts was challenging. Baş et al. reported a single case with successful SCEP recording, also describing two peaks: P1 (latency 5-10 ms) and P2 (latency ∼40 ms), markedly different from the findings of Boëx et al. and Zauli et al. (Baş et al., 2023). In the provided figure, peaks identification and reproducibility remain unclear.

Overall, SCEP recording appears technically feasible. However, despite promising preliminary findings, substantial heterogeneity exists in both methodology and results. More structured studies are needed to establish reproducibility, define more standardized methodology and, finally, assess potential clinical validation.

#### 4.2.2 Subcortical recorded visual evoked potentials (sVEP)

Although sVEPs and conventional VEPs may appear technically similar and differ mainly in the site of recording, this distinction fundamentally changes their intraoperative role. Conventional VEPs are used as a monitoring technique to assess visual pathway function during surgery, whereas sVEPs are intended as a focal mapping technique, in which subcortical recordings are obtained intermittently at selected sites to assess local proximity to the optic radiation. This site-specific and intermittent assessment is inherent to intraoperative mapping techniques, including direct subcortical stimulation in an awake setting, which are performed only at the surgical site being explored.

sVEP recording was first described by Shahar et al., in 2017 (Shahar et al., 2017). Using conventional flash VEP stimulation (pulse width 15 μs, frequency 1.2 Hz), they attempted to record subcortical responses within the OR in 13 patients, obtaining positive responses in 10 cases. Recordings were performed using a monopolar probe at the depth of the surgical cavity with a reference electrode at FPz, with a bandwidth of 1–300 Hz. Subcortical recordings demonstrated reproducible waveforms resembling cortical VEPs, with an N2 peak around 60-80 ms and a P2 peak around 90-120 ms. Notably, stimulation sites located farther from the OR did not yield responses.

In 2024, Zauli et al. replicated sVEP recordings in two epilepsy patients using a 4-contact SEEG electrode inserted in the white matter targeting the OR with neuronavigation (Zauli et al., 2024), showing a moderate reproducibility between two acquisitions. Waveforms differed significantly from those reported by Shahar et al., consisting of an initial small positive deflection (latencies of 50.2 ms and 57.7 ms in the two cases), followed by a burst of high-frequency oscillations (100–150 Hz). Differences between the two articles may partly reflect variations in recording configuration, monopolar vs bipolar and especially the electrode surface.

Shahar et al. correlated postoperative MRI measurements with intraoperative findings, showing that sVEP responses were consistently present when the distance between the bottom of the surgical cavity and the OR, as estimated by DTI, was <10 mm (except in one case with absent baseline VEP) (Shahar et al., 2017). In contrast, Zauli et al. estimated this distance intraoperatively by measuring the distance between the recording contact inserted in the white matter (assumed to be located within the OR by neuronavigation) and the intraparenchymal contact closest to the bottom of the surgical cavity (Zauli et al., 2024).

Similarly to SCEPs, sVEP recording appears technically feasible. However, despite internal consistency within individual studies, substantial methodological and neurophysiological variability exists between studies, and the number of included patients remains limited. Larger, standardized investigations are required to define optimal protocols, assess reproducibility, and determine clinical relevance.

### 4.3 Awake mapping of the OR

Duffau et al. first reported subcortical stimulation of the OR in 2004 during low-grade glioma awake surgery (Duffau et al., 2004). Penfield stimulation near the OR induced errors during a picture-naming task, associated with visual disturbances such as shadows in the contralateral superior visual field and image distortion.

#### 4.3.1 The modified picture-naming task

In 2012 Gras-Combe et al. (2012) reported the first case series and introduced a modified picture-naming task specifically designed for OR mapping. This task involved the simultaneous presentation of two images positioned diagonally on a screen, with one image displayed in the visual quadrant intended to be preserved and the other in the opposite quadrant. Patients were asked intraoperatively to maintain fixation on a central cross while continuously naming the images presented in successive pairs on the screen, allowing differentiation between language impairment and visual field disturbances. Stimulation was performed in a blinded fashion, using a bipolar probe and following the Penfield paradigm (biphasic stimulation, frequency 60 Hz, single pulse, pulse width 1 ms, intensities ranging from 1.5 to 6 mA across studies).

Table 3 summarizes the clinical outcome of patients who performed a modified-picture naming task. As previously mentioned, at follow-up 23% of patients presented no postoperative visual deficits, 68% developed an (expected) quadrantanopia, and 8% developed homonymous hemianopia.

From a functional standpoint, homonymous hemianopia is generally incompatible with driving in most countries, whereas quadrantanopia, particularly superior, may be acceptable in many cases (Kobal and Hawlina, 2022; Yan et al., 2019; Bron et al., 2010). Since maximal safe resection represents the primary goal of neurooncological surgery, the modified picture-naming task has been demonstrated to be an effective tool to minimize major visual deficits.

It should be noted that the majority of reported cases involved low-grade gliomas (75 versus 35 high-grade gliomas), and clinical outcomes were not systematically differentiated by tumor grade, reducing the generalizability of this mapping strategy to high-grade glioma surgery.

#### 4.3.2 Alternative tasks for awake mapping of the OR

Nguyen et al. (2011) proposed a combination of static and kinetic perimetry using a screen with red targets on half corresponding to the at-risk visual field, green targets on the opposite side and a central fixation cross. During the static test, the patient reported any fading or disappearance of the targets, whereas kinetic testing was performed by moving a laser spot from the periphery toward the center and across the vertical meridian. Similarly, Šteňo et al. (Šteňo et al., 2012; Šteňo et al., 2014) displayed a single figure of a dog on the screen with a central fixation cross. The operator used a laser pointer to indicate different parts of the image and asked for verbal confirmation of their localization. In one case, stimulation was also performed with the patient's eyes covered, resulting in more pronounced phosphenes.

Although it remained unclear whether dynamic testing provided additional information compared with static testing, Nguyen et al. were the only authors to propose this approach (Nguyen et al., 2011). In contrast, Šteňo et al. proposed a flexible and adaptive paradigm (Šteňo et al., 2012; Šteňo et al., 2014), but also less standardized and potentially less reproducible; furthermore, they explored stimulation with eyes covered as a means of enhancing sensitivity (Šteňo et al., 2014).

In subsequent years, Conti Nibali et al. (Conti Nibali et al., 2020) introduced the intraoperative visual field task (iVT), comparing it with the modified picture-naming task. The iVT consisted of the presentation of three different target numbers appearing randomly one at a time for 0.3-0.5 s in the peripheral visual field of a tablet screen at different vertical positions. Compared with the modified picture-naming task, the iVT was associated with a lower incidence of quadrantanopia (33% vs 63%), and with no cases of symptomatic quadrantanopia (vs 32% with modified picture-naming task) or homonymous hemianopia (vs 10%), with a comparable extent of resection between groups. Finally, Mazerand et al. (2017) reported an intraoperative application of a modified Esterman test using a virtual reality headset (FEX-VRH, Functions’ Explorer Virtual Reality Headset). During stimulation, luminous stimuli were delivered and the patient was asked to report their perception. The position of the stimuli in the visual field was selected on a separate interface, and resection was halted upon detection of a hemianopia during stimulation.

Conti Nibali et al. (Conti Nibali et al., 2020) were the only authors to perform a comparative analysis, suggesting a potential advantage of the iVT over standard testing. These results are promising; however, further validation and assessment of feasibility across different centers are required. Although virtual reality-based approaches are increasingly being introduced into clinical practice and have shown encouraging preliminary results (Mazerand et al., 2017), their superiority over simpler and more accessible paradigms remains to be demonstrated before routine clinical implementation.

### 4.4 Implications for future research

Awake mapping using the modified picture-naming task represents the current reference standard, supported by the largest body of evidence and available clinical outcome data, although predominantly performed in low-grade glioma cases. Alternative approaches, particularly the iVT, may offer improved functional outcomes; however, further studies are required to confirm these findings and to assess their feasibility across different clinical settings. Future research should explore dynamic testing paradigms, task personalization, and the integration of advanced technologies. In particular, virtual reality and automated task delivery may play an increasing role, given ongoing technological advancements and their expanding clinical applicability.

Evoked potential recordings of white matter tracts represent a rapidly evolving field, enabling monitoring and mapping of association and projection fibers during surgery under general anesthesia, since awake surgery may not be feasible in all patients. These approaches also provide objective and quantifiable readouts in the form of neurophysiological waveforms. The two modalities described in the literature appear conceptually coherent and technically feasible; however, substantial heterogeneity exists in both methodology and reported results, and their clinical validity remains to be established.

Future prospective studies should therefore adopt a structured reporting framework, potentially adapted from recent STARD-IONM recommendations (Thirumala et al., 2025). Prespecified study protocols should be made available, or methodological descriptions should be sufficiently detailed to allow replication. Reporting should include anesthetic technique and depth, neuromuscular blockade, and intraoperative physiological management. Studies should also use standardized preoperative and postoperative assessment of visual function, preferably including formal visual field testing, clinical assessment, and validated patient-reported outcome measures. Assessment time points should be predefined and include an early postoperative evaluation, assessment at discharge, and late follow-up, for example at 3 months, to distinguish transient from persistent visual deficits. Finally, the handling of missing data should be explicitly reported.

### 4.5 Limitations

This review has several limitations. First, the limited number of studies and patients precluded meaningful comparison between techniques. Second, heterogeneity in study design and reporting prevented a comprehensive analysis of clinical outcomes. Third, the predominance of low-grade glioma cases limits the applicability of the available evidence to high-grade glioma surgery, where awake craniotomy could present additional technical challenges and require a more individualized risk-benefit assessment.

A formal risk-of-bias assessment was not performed, as it fell beyond the scope of this review. Nevertheless, the overall strength of the evidence appears limited, particularly for evoked potential recording techniques. Many included papers were case reports or small single-center series, often with incomplete methodological descriptions, heterogeneous outcome assessment, and limited reporting of clinically relevant results. In studies of evoked-potential recording, technical details such as recording configuration, waveform characteristics, and consistency of findings across patients were frequently insufficiently described, while the small cohorts precluded robust assessment of reproducibility. These studies were nevertheless included to provide a comprehensive overview of existing techniques, organize the available evidence, and identify directions for future standardization and clinical investigation.

## 5 Conclusions

Awake mapping using the modified picture-naming task has established a solid foundation for the intraoperative identification and preservation of the optic radiation during low-grade glioma surgery. Alternative awake behavioral paradigms represent promising refinements that warrant further investigation, as they may broaden the range of functions that can be monitored intraoperatively and allow better adaptation to individual patient profiles; however, robust clinical validation is still lacking.

Evoked potential recording approaches represent a qualitatively different step forward, as they can provide an objective, waveform-based correlate of optic radiation function, applicable even in patients operated under general anesthesia in whom awake surgery is not feasible. This makes them particularly compelling, both as clinical tools and as research instruments to further characterize the functional anatomy of white matter pathways. However, standardization and reproducibility remain the key requirements to enable investigation of clinical validity, before any eventual translation into clinical practice.

## Author contributions

M.O., P.A.-A., and K.S. conceived the study. M.O. performed data collection and analysis and wrote the first draft of the manuscript. P.A.-A. and S.S. contributed to data analysis and manuscript editing. A.R. and P.S. provided supervision and critical revision. K.S. supervised the study, resolved discrepancies, and contributed to manuscript editing and final revision. All authors reviewed and approved the final version of the manuscript.

## Funding

None.

## Declaration of competing interest

The authors declare that they have no known competing financial interests or personal relationships that could have appeared to influence the work reported in this paper.
