# Supplementary material for: Subcortical brain mapping strategies for intraoperative identification of the optic radiation during asleep and awake surgery: a scoping review
Source: Brain Spine. 2026 Jul 14;6:106175. doi: 10.1016/j.bas.2026.106175 (PMC13382403; doi:10.1016/j.bas.2026.106175)
Supplement: Multimedia component 1 [file mmc1.docx]

**Supplementary material**

1. **Search strategy**

|  |  | Pubmed | Scopus | Embase | EBSCO | Cochrane Library | Overall |
| --- | --- | --- | --- | --- | --- | --- | --- |
| Research 1 | ("Optic radiation" OR (visual AND pathway)) AND (mapping OR recording) AND glioma | 134 | 73 | 46 | 51 | 7 | 311 |
| Research 2 | subcortical stimulation optic tract | 38 | 76 | 47 | 44 | 1 | 206 |
| Research 3 | Axono-cortical evoked potentials | 12 | 13 | 15 | 12 | 0 | 52 |
| Research 4 | subcortical mapping optic (radiation OR tract) | 77 | 66 | 43 | 58 | 0 | 244 |
| Research 5 | Subcortico-cortical evoked potentials | 17 | 24 | 16 | 19 | 1 | 77 |
| Research 6 | ("Optic radiation" OR (visual AND pathway)) AND intraoperative AND (mapping OR recording) AND (tumor) | 95 | 54 | 18 | 37 | 1 | 205 |
| Total | | 373 | 306 | 185 | 221 | 10 | 1095 |

1. **Optic pathways – 3D model**

The simplified interactive three-dimensional (3D) model representing the optic pathways in relationship to the ventricular system and the cerebral cortex is publicly available on the 3D media repository Sketchfab (Sketchfab Inc., New York, NY, USA) at the following link: [https://skfb.ly/pIVrx](https://skfb.ly/pIVrx). The model was created on data from the Human Connectome Project (WU-Minn Consortium; PIs: Van Essen and Ugurbil; subject ID: 100307). Tractography was performed using DSI Studio (Hou version; developed by Fang-Cheng Yeh, University of Pittsburgh, Pittsburgh, PA, USA) with generalized q-sampling imaging (GQI) reconstruction and orientation distribution function (ODF)-based deterministic fiber tracking. Volume segmentations were performed using 3D Slicer 5.8 (Brigham and Women’s Hospital, Boston, MA, USA). The 3D model was then integrated and rendered using Blender 4.4 (Blender Foundation, Amsterdam, the Netherlands).

1. **Corkscrew recording montages during subcortico-cortical evoked potentials**

| **Figures** | Boëx et al. (1) | Boëx et al. (2) | Baş et al. |
| --- | --- | --- | --- |
| **Proposed montage** | O1-Cz, O2-Cz, PO7-Cz, PO8-Cz, P4-Cz, Cp3-Cz, Cp4-Cz | Cp4-Cp3, Cpz-Fz, O1-Fz, P7-Fz, PT3-Fz, O2-Fz, P8-Fz, PT4-Fz | O1-A1, O2-A2, Oz-Cz, O1-Cz, O2-Cz, P7-Cz, T7-Cz, F7-Cz, P8-Cz, T8-Cz, F8-Cz |
